# Transcriptomics integrated with metabolomics reveals partial molecular mechanisms of nutritional risk and neurodevelopment in children with congenital heart disease

**DOI:** 10.3389/fcvm.2024.1414089

**Published:** 2024-08-09

**Authors:** Minglei Gao, Yang Shen, Ping Yang, Chang Yuan, Yanan Sun, Zipu Li

**Affiliations:** ^1^ Heart Center, Qingdao Women and Children’s Hospital, Shandong University, Qingdao, China; ^2^ Heart Center, Dalian Women and Children’s Medical Group, Dalian, China; ^3^ Clinical Laboratory, Dalian Women and Children’s Medical Group, Dalian, China

**Keywords:** congenital heart disease, nutritional risk, neurodevelopment, transcriptomics, metabolomics

## Abstract

**Purpose:**

To explore molecular mechanisms affecting nutritional risk and neurodevelopment in children with congenital heart disease (CHD) by combining transcriptome and metabolome analysis.

**Methods:**

A total of 26 blood and serum samples from 3 groups of children with CHD low nutritional risk combined with normal neurodevelopment (group A), low nutritional risk combined with neurodevelopmental disorders (group B) and high nutritional risk combined with normal neurodevelopment (group C) were analyzed by transcriptome and metabolomics to search for differentially expressed genes (DEGs) and metabolites (DEMs). Functional analysis was conducted for DEGs and DEMs. Further, the joint pathway analysis and correlation analysis of DEGs and DEMs were performed.

**Results:**

A total of 362 and 1,351 DEGs were detected in group B and C compared to A, respectively. A total of 6 and 7 DEMs were detected in group B and C compared to A in positive mode, respectively. There were 39 and 31 DEMs in group B and C compared to A in negative mode. Transcriptomic analysis indicated that neurodevelopment may be regulated by some genes such as NSUN7, SLC6A8, CXCL1 and LCN8, nutritional risk may be regulated by SLC1A3 and LCN8. Metabolome analysis and joint pathway analysis showed that tryptophan metabolism, linoleic and metabolism and glycerophospholipid metabolism may be related to neurodevelopment, and glycerophospholipid metabolism pathway may be related to nutritional risk.

**Conclusion:**

By integrating transcriptome and metabolome analyses, this study revealed key genes and metabolites associated with nutritional risk and neurodevelopment in children with CHD, as well as significantly altered pathways. It has important clinical translational significance.

## 1 Introduction

Congenital heart disease (CHD) is a congenital heart malformation caused by abnormal development of fetal heart and large blood vessels, and is the most common heart disease in children (1, 2). CHD is the most common birth defect, and prevalence of CHD in live born infants is 1%–1.2% (1, 3). Due to the variety, complexity and difficulty in treating CHD, its mortality is on the rise in China (4). CHD increases the risk of neurodevelopmental disorders, including cognitive, motor, social adjustment, and behavior disorders (5–7). Neurodevelopmental delays may reduce the lifespan of children with CHD and cause cognitive or intellectual impairment (8). This not only increase the risk of worse prognosis, but also has serious impact on the entire life, including academic performance, employment opportunities, psychological and overall quality of life (8, 9). Hence, it is essential to explore the molecular mechanism of neurodevelopment in children with CHD and the interventions targeted at the early stage of the development to improve the quality of life. Growth failure is also a common problem in children with CHD (10, 11). The etiology and pathological mechanism of slow physical and mental development in infants with CHD remain unclear, but there are many risk factors associated with it, including severity of cyanosis, chronic hypoxia, hemodynamic changes, repeated infections and heart failure, repeated hospitalization, and feeding disorders (11, 12). Nutrition, parents’ education level, living environment, social and family environment, living style and economic level will also affect the physical and intellectual development of children with CHD (11, 13). Perioperative young children required more caloric and nutrient intake to promote adequate growth and psychomotor development, and energy and protein deficiencies can increase infections and inflammatory responses, impair wound healing, prolong hospital stays, and may increase the incidence of postoperative complications (14, 15). Therefore, optimal nutrition is crucial to improve the short- and long-term prognosis of children with CHD.

Omics analysis techniques are increasingly used to identify potential biomarkers and elucidate causes and mechanisms associated with disease (16, 17). Metabolomics can analysis small molecule metabolites to reflect the biological metabolic characteristics of disease states and is used extensively in biomarker discovery (18). Dong S et al. analyzed the heart's metabolic remodeling to hypoxia using metabolomics and found protein synthesis and aerobic energy production were reduced in patients with cyanotic CHD and NAD may play an important role in response to hypoxia (19). Jin N et al. found S-Adenosyl methionine, guanine and N-terminal pro-brain natriuretic peptide could be used as biomarkers for pulmonary arterial hypertension associated with CHD from CHD (20). Transcriptome research can study gene function and gene structure at the whole level and reveal the molecular mechanism of specific biological processes and disease occurrence (21). Liu G et al. identified genes and enriched pathways associated with radiotherapy in nasopharyngeal carcinoma, revealing the molecular mechanism of radiotherapy resistance, which is helpful for future research on radiotherapy resistance function (22). The combined transcriptomics and metabolomic analysis of high-risk neuroblastoma and low and intermediate-risk neuroblastoma identified 4 aberrant pathways and developed a risk classification diagnostic model, providing insights for high-risk neuroblastoma early diagnosis (23). Therefore, transcriptomics and metabolomics play an important role in diagnosis of diseases, and their molecular mechanisms can be deeply explored.

In this study, transcriptomic and metabolomic analysis of blood and serum samples from 26 children with CHD was performed to search for key DEGs and DEMs, and explore molecular mechanisms affecting nutritional risk and neurodevelopment in children with CHD.

## 2 Methods and materials

### 2.1 Sample collection

A total of 26 blood and serum samples were collected from hospitalized children with CHD in the Dalian Children's Hospital. Blood samples (2 ml/sample) were used for transcriptomic analysis and serum samples (1 ml/sample) were used for metabolomics analysis. Main inclusion criteria: (1) confirmed diagnosis of CHD; (2) aged 0–24 months; (3) written informed consent was obtained from child's guardians. Main exclusion criteria: (1) children with nutritional impairments due to major non-cardiac diseases; (2) multiple pregnancy; (3) chromosomal abnormality; (4) structural brain malformation; (5) placental dysfunction; (6) intrauterine growth retardation of the fetus; (7) received the prescribed nutrition supply and blood transfusion half a month before hospitalization. Blood and serum samples were collected 24 h before discharge from the hospital (or after surgery). The children with CHD were divided into three groups according to the nutritional risk and neurodevelopmental status: low nutritional risk combined with normal neurodevelopment (*n* = 14, group A), low nutritional risk combined with neurodevelopmental disorders (*n* = 5, group B), high nutritional risk combined with normal neurodevelopment (*n* = 7, group C). The screen tool for risk on nutritional status and growth (STRONGkids) was used for nutritional risk screening and nutritional assessment of hospitalized children over 1 month. The Revised Gesell Developmental Schedules were used to assess the neurodevelopmental status of CHD children aged 6 months to 24 months. This study was approved by the Ethics Committee of Dalian Children's Hospital (19015).

### 2.2 Transcriptomics detection through RNA-Sequencing (RNA-Seq) analysis

Total RNA was extracted from the samples with TRIzol. Sequencing By Synthesis (SBS) technology was used to sequence the cDNA library using Illumina's high-throughput sequencing platform. The raw data obtained by sequencing were converted into FASTQ format sequence data by base calling. The raw data was analyzed using a bioinformatic workflow, which includes quality control with MultiQC, adapter and quality trimming with Cutadapt, transcript quantification with featureCounts (GRCh38.primary_assembly.genome.fa). Differential expression analysis was performed using the DESeq2 in R package (4.0.5) (*p* value < 0.05 and |log_2_foldchange| > 1). The Gene Ontology (GO) and Kyoto Encyclopedia of Genes and Genomes (KEGG) function enrichment analysis of differentially expressed genes (DEGs) were performed using the David database (https://david.ncifcrf.gov/tools.jsp).

### 2.3 Non-targeted metabolomics analysis via liquid chromatograph with tandem mass spectrometer (Lc–Ms/Ms)

The 300 μl acetonitrile was added to 100 μl serum at 4℃. After 20 min of centrifugation at 12,000 r/min (4℃), 100 μl supernatant was carefully extracted. Take 50 μl as quality control (QC) samples. All of the extracts were analyzed using LC–MS/MS technology with a high performance liquid chromatography (HPLC) and a high resolution mass spectrometer (HRMS). The chromatographic separation was performed on a Waters HSS T3 column. Metabolomics data were collected as follows: a column temperature of 45°C and the flow rate of 0.3 ml/min. mobile phase: A = 0.1% formic acid water, B = 0.1% formic acid acetonitrile. Mass spectrometry (MS) negative and positive mode conditions: sheath gas flow rates 30 arb, atomization pressure 50 psi. capillary voltage: positive mode 5,500 V, negative mode −4,500 V. Based on the MS detection, the original files were imported into Progenesis QI (Waters) software for data pre-processing and identification, and then the data quality control analysis was carried out to ensure the accuracy and reliability of the data. Multivariate statistical analysis was performed on the data to reveal the differences of metabolites of different components, the biological significance of metabolites was discovered through functional analysis. Orthogonal partial least squares discrimination analysis (OPLS-DA) was used to filter signals that are not relevant to classification. Potential metabolites were analyzed according to predicted value (VIP) and significance of variables in Student's *t*-test. VIP > 1 and *p *< 0.05 were considered as statistically significant. Differentially expressed metabolites (DEMs) were selected using KEGG (https://www.genome.jp/kegg/) and human metabolome database (HMDB) (https://hmdb.ca/), and further annotated in the KEGG com-pound database. The annotated metabolic pathways were classified according to the KEGG pathway database (https://www.kegg.jp/kegg/pathway.html).

### 2.4 Joint analysis of the metabolomics and transcriptomics

Combined pathway analysis of DEMs and DEGs were performed using MetaboAnalyst 6.0 (https://www.metaboanalyst.ca/). The cor.test function in R software was used to calculate Pearson correlation coefficient to construct the correlation analysis between the above metabolites and genes.

## 3 Results

### 3.1 Transcriptome sequencing analysis and identification of DEGs

To explore the correlation between nutritional risk and neurodevelopment in children with CHD, we performed transcriptomic analysis of the three groups. Principal component analysis (PCA) was used to test the reliability of the experiment and the rationality of sample selection. Pearson correlation coefficient, *R*^2 ^> 0.8 was considered as good repeatability sample. As shown in Supplementary Figure S1, samples within the group have good repeatability, and samples between the groups have large differences. Sample N14 was significantly different from other samples, so it was excluded in the subsequent analysis.

**Figure 1:**
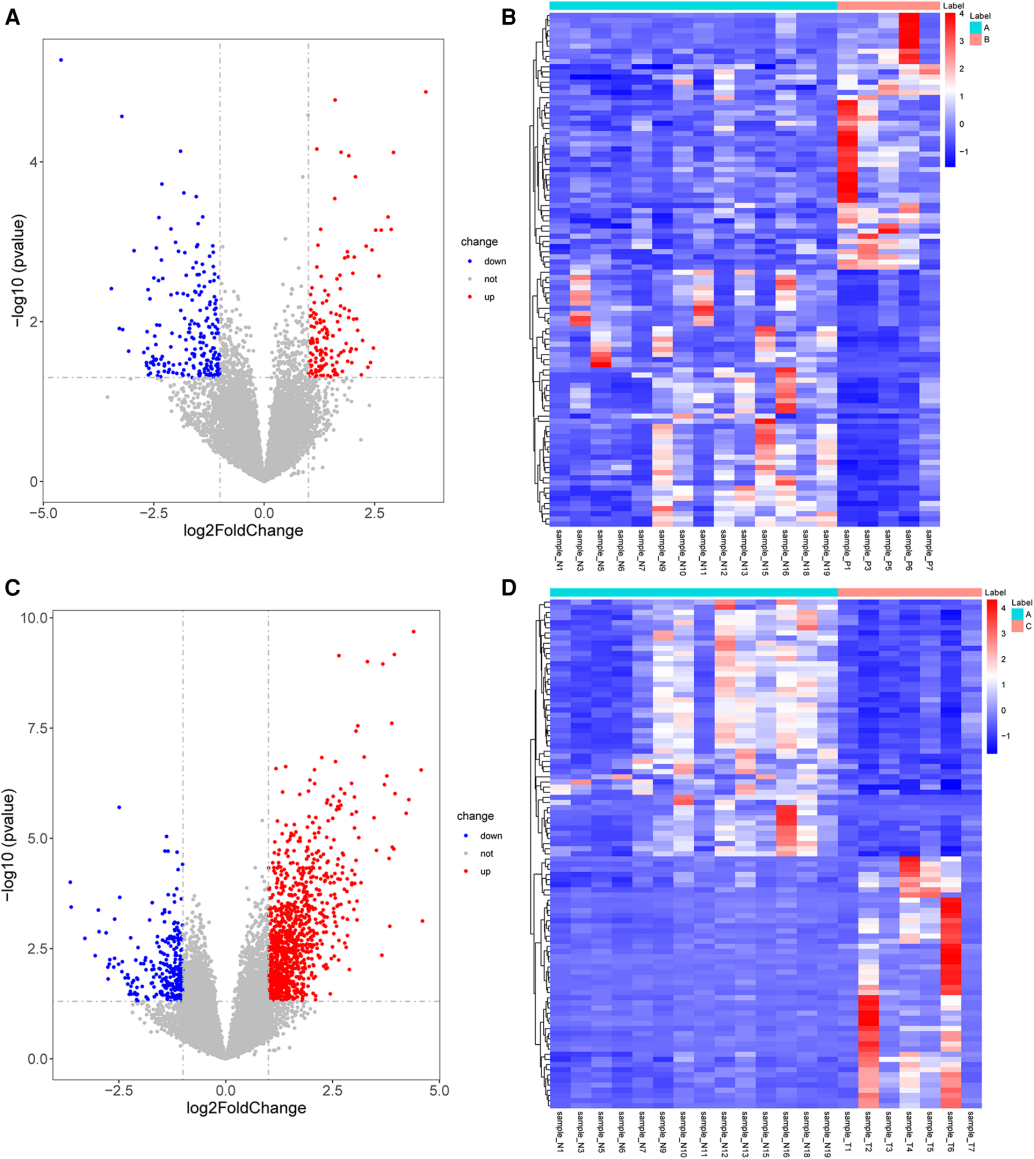
Identification of DEGs. (**A**) The volcano plot shows DEGs between group B and A. (**B**) The heatmap shows DEGs between group B and A. (**C**) The volcano plot shows DEGs between group C and A. (**D**) The heatmap shows DEGs between group C and A. DEGs, differentially expressed genes.

A total of 362 DEGs were screened in group B compared to A, of which 145 up-regulated genes and 217 down-regulated genes were detected (Figures 1A,B). The top 20 up-regulated and down-regulated genes in group B compared to A are shown in Table 1. A total of 1,351 DEGs were screened in group C compared to A, of which 1,079 up-regulated genes and 272 down-regulated genes were detected (Figures 1C,D). The top 20 up-regulated and down-regulated genes in group C compared to A are shown in Table 2.

**Table 1:** The top 20 up-regulated and down-regulated genes in group B compared to A.

Gene	log_2_foldchange	*P* value	Regulation
XKR3	3.660188629	1.34 × 10^−05^	Up
NSUN7	1.605486283	1.69 × 10^−05^	Up
TNFRSF12A	1.192398703	6.92 × 10^−05^	Up
VSIG4	1.740256735	7.59 × 10^−05^	Up
RXFP2	2.926853126	7.62 × 10^−05^	Up
CXCL1	1.915904565	8.39 × 10^−05^	Up
KIAA0319	2.066134462	0.000153784	Up
FAM118A	1.597664361	0.000288011	Up
H2BC17	2.805629365	0.000489592	Up
SPACA6	1.279218851	0.000699556	Up
LCN8	−4.599140337	5.32 × 10^−06^	Down
RAP1GAP	−3.222966552	2.70 × 10^−05^	Down
SLC6A8	−1.89378474	7.36 × 10^−05^	Down
TUBB2A	−2.316078348	0.000189687	Down
SLC4A1	−1.818465674	0.000244753	Down
AQP1	−1.537989591	0.000272528	Down
AGRN	−1.391133482	0.000488191	Down
SPTB	−2.383261022	0.00049809	Down
DMTN	−1.495462112	0.000593119	Down
MX1	−2.111932078	0.000692207	Down

**Table 2:** The top 20 up-regulated and down-regulated genes in group C compared to A.

Gene	log_2_foldchange	*P* value	Regulation
SLC1A3	4.39683	2.06 × 10^−10^	Up
IL18R1	3.94469	6.79 × 10^−10^	Up
FKBP5	2.64747	7.20 × 10^−10^	Up
PFKFB2	3.31390	9.84 × 10^−10^	Up
ECHDC3	3.67517	1.12 × 10^−09^	Up
IL1R2	3.88479	2.46 × 10^−08^	Up
SOCS3	3.09022	2.81 × 10^−08^	Up
SIPA1L2	3.04663	3.70 × 10^−08^	Up
IL18RAP	3.23829	1.43 × 10^−07^	Up
FPR2	2.24746	1.47 × 10^−07^	Up
KCNG1	−2.49326	1.99 × 10^−06^	Down
IL32	−1.38511	9.10 × 10^−06^	Down
C2orf92	−1.42335	1.95 × 10^−05^	Down
RTN4R	−1.35111	1.96 × 10^−05^	Down
NAA80	−1.13880	2.07 × 10^−05^	Down
BCAS4	−1.00919	3.88 × 10^−05^	Down
NPIPB15	−1.11527	5.12 × 10^−05^	Down
LCN8	−3.63616	9.89 × 10^−05^	Down
LYPD3	−1.14690	0.000139592	Down
SPATC1l	−1.34686	0.000190124	Down

### 3.2 GO analysis and KEGG pathway analysis

**Figure 2:**
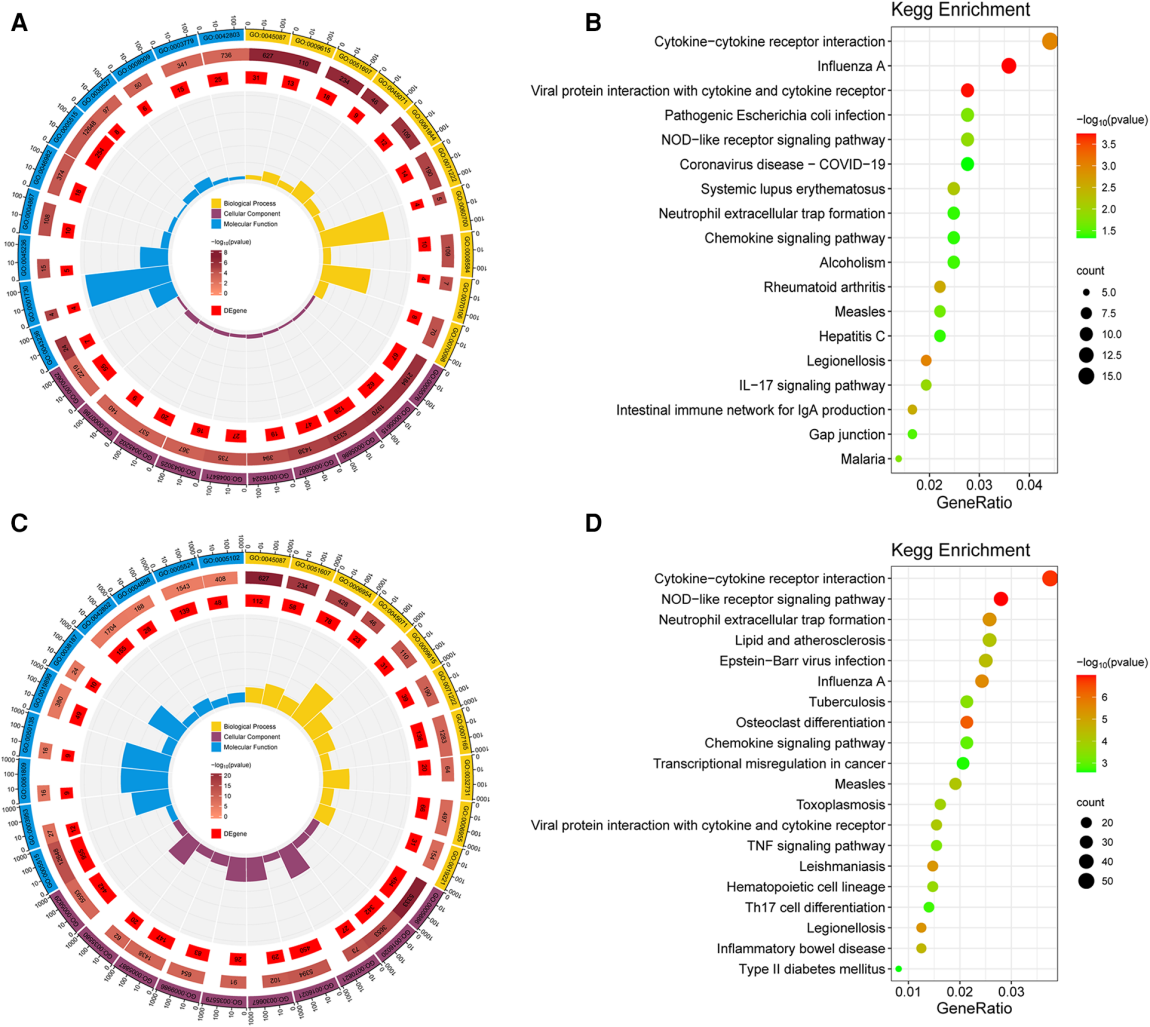
Go and KEGG analysis of DEGs. (**A**) GO analysis of biological processes, cellular components and molecular functions of DEGs between group B and A. (**B**) Pathway enrichment of the DEGs between group B and A. (**C**) GO analysis between group C and A. (**D**) Pathway enrichment of the DEGs between group C and A. The dot color represents the *p*-value and the dot size represents the number of DEGs. DEGs, differentially expressed genes.

GO and KEGG enrichment analysis of the obtained DEGs was performed to analyze the key functions and metabolic pathways (Figure 2; Supplementary Table S1). The top three significantly enriched terms were innate immune response, response to virus and defense response to virus in biological processes in group B compared with group A. The top three significantly enriched terms were extracellular region, extracellular space and plasma membrane in cellular component in group B compared with group A. The top three significantly enriched terms were laminin binding, oligoadenylate synthetase activity and CXCR chemokine receptor binding in molecular function in group B compared with group A (Figure 2A). The top three significantly enriched terms were innate immune response, defense response to virus and inflammatory response in biological processes in group C compared with group A. The top three significantly enriched terms were plasma membrane, membrane and tertiary granule membrane in group C compared with group A. The top three significantly enriched terms were protein binding, NAD^+^ nucleosidase activity and NAD^+^ nucleotidase, cyclic ADP-ribose generating in molecular function in group C compared with group A (Figure 2C). KEGG analysis found that the top three altered pathways were influenza A, viral protein interaction with cytokine and cytokine receptor and cytokine-cytokine receptor interaction in group B compared with group A (Figure 2B), and the top three altered pathways were NOD-like receptor signaling pathway, cytokine-cytokine receptor interaction and osteoclast differentiation in group C compared with group A (Figure 2D). In summary, compared with group A, the key functions of both groups B and C are significantly enriched innate immune response and defense response to virus and plasma membrane, and the significant enrichment pathway is cytokine-cytokine receptor interaction.Metabolomics analysis and identification of DEMs.

Correlation analysis of the QC samples showed that *R*^2^ values were greater than 0.8 (Supplementary Figures 2A,B), indicating that the entire analysis process was stable and reproducible. OPLS-DA was performed to screen the reliable metabolites that lead to the classification difference. The results showed that the metabolites of the two groups with low nutritional risk combined with neurodevelopmental disorders and high nutritional risk combined with normal neurodevelopment were significantly different from those of the healthy control group in both positive and negative modes (Supplementary Figures 2C–F).

**Figure 3:**
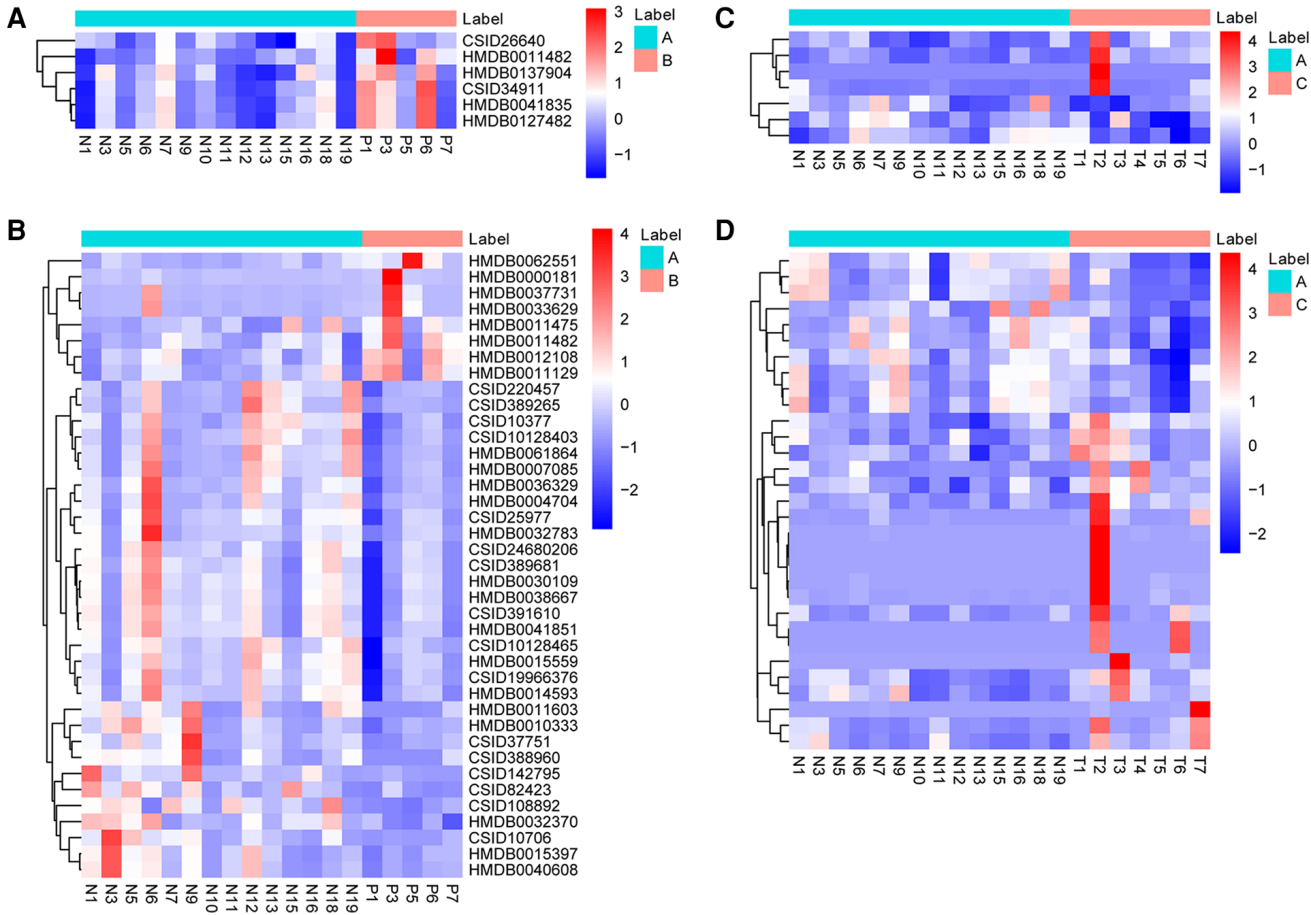
Identification of DEMs. (**A**) The heatmap shows DEMs in positive mode between group B and A. (**B**) The heatmap shows DEMs in negative mode between group B and A. (**C**) The heatmap shows DEMs in positive mode between group C and A. (**D**) The heatmap shows DEMs in negative mode between group C and A. DEMs, differentially expressed metabolites.

Metabolites with VIP > 1 and *p *< 0.05 in QC samples were selected as DEMs. In positive mode, 6 DEMs were identified in group B compared to A, all of which were up-regulated metabolites (Figure 3A; Table 3). A total of 39 metabolites changed significantly in negative mode in group B compared to A, including 8 up-regulated and 31 down-regulated metabolites (Figure 3B; Table 4). Similarly, compared with A, we found a total of 7 DEMs in positive mode in group C, 4 of which were up-regulated and 3 down-regulated (Figure 3C; Table 3). In negative mode, 31 DEMs were identified in group C compared to A, including 21 up-regulated and 10 down-regulated metabolites (Figure 3D; Table 4).

**Table 3:** Differentially expressed metabolites in B vs. A and C vs. A in positive mode.

	ID	Metabolites	Mass-to-charge ratio	Retention time (min)	VIP value	Fold change	*P* value	Regulation
B vs. A	HMDB0011482	LysoPE[0:0/20:1 (11Z)]	508.339	9.83	1.1446	1.732	0.0361307	Up
	CSID34911	Flunixin	297.082	9.84	1.2712	1.546	0.0319379	Up
	HMDB0041835	Benzidine	223.063	9.83	1.4013	1.497	0.0461483	Up
	HMDB0137904	4-hydroxy-2H-chromen-2-one	163.038	7.14	1.6213	1.430	0.0317435	Up
	HMDB0127482	6-[(2-carboxyacetyl)oxy]-3,4,5-trihydroxyoxane-2-carboxylic acid	281.050	9.83	1.1966	1.409	0.0469276	Up
	CSID26640	S-acetylcysteamine	239.088	13.43	1.4357	1.319	0.0128131	Up
C vs. A	HMDB0029581	(2E,4E)-2,4-Hexadienoic acid	135.043	1.13	1.4834	142.146	0.0458151	Up
	CSID2298569	J4CLF34O60	633.254	7.80	1.0549	2.752	0.0394431	Up
	HMDB0005065	Oleoylcarnitine	426.357	9.87	4.1512	1.512	0.0094583	Up
	CSID391217	3-{[(3aS,7aR)-2-Hydroxyoctahydrofuro[3,2-b]pyridin-2-yl]methyl}-4 (3H)-quinazolinone	603.292	10.47	1.0548	1.389	0.0471863	Up
	HMDB0010408	LysoPC[P-18:1 (9Z)]	506.363	11.43	1.2934	0.725	0.0270095	Down
	HMDB0010393	LysoPC[20:3 (5Z,8Z,11Z)]	546.355	10.11	3.5653	0.656	0.0468882	Down
	HMDB0010581	PG[16:0/22:4 (7Z,10Z,13Z,16Z)]	799.548	13.44	2.3672	0.608	0.0351869	Down

**Table 4:** Differentially expressed metabolites in B vs. A and C vs. A in negative mode.

	ID	Metabolites	Mass-to-charge ratio	Retention time (min)	VIP value	Fold change	*P* value	Regulation
B vs. A	HMDB0000181	L-Dopa	178.050	4.13	3.0002	10.822	0.0264023	Up
	HMDB0062551	4-ethylphenylsulfate	201.021	5.64	1.6227	5.595	0.0105276	Up
	HMDB0037731	S-(2-Furanylmethyl) methanethioate	187.007	4.77	6.6878	4.175	0.0375045	Up
	HMDB0033629	xi-4-Hydroxy-4-methyl-2-cyclohexen-1-one	107.049	4.77	1.6498	3.775	0.0389009	Up
	HMDB0012108	LysoPC(17:0)	554.346	10.58	4.5436	1.675	0.0414659	Up
	HMDB0011482	LysoPE[0:0/20:1 (11Z)]	552.330	9.81	2.4413	1.529	0.0225322	Up
	HMDB0011129	LysoPE(0:0/18:0)	526.314	9.46	4.7880	1.523	0.0378447	Up
	HMDB0011475	LysoPE[0:0/18:1 (11Z)]	478.293	9.24	2.0505	1.393	0.0497672	Up
	CSID108892	zoxamide	669.036	12.80	1.0700	0.783	0.0302921	Down
	CSID10128465	6alpha-Fluorotestosterone acetate	393.213	13.58	2.1775	0.735	0.0408695	Down
	HMDB0032370	L-Menthyl (R,S)-3-hydroxybutyrate	241.180	12.56	1.1991	0.731	0.0022804	Down
	HMDB0015559	Thioproperazine	427.163	13.59	3.9359	0.721	0.0338609	Down
	HMDB0030109	Albanol B	557.120	13.60	1.7918	0.703	0.0442844	Down
	CSID19966376	Bendazac L-lysine	409.190	13.59	4.9536	0.676	0.0273167	Down
	CSID10377	Benzoyl-y-tropeine	489.277	13.51	2.4271	0.674	0.0219379	Down
	HMDB0038667	N,N'-Bis(g-glutamyl)-3,3'-(1,2-propylenedithio)dialanine	539.148	13.60	2.2162	0.673	0.0327456	Down
	CSID389681	5'-O-[Hydroxy({5-[(4S)-2-oxohexahydro-1H-thieno[3,4-d]imidazol-4-yl]pentanoyl}oxy)phosphoryl]adenosine	554.123	13.60	2.0477	0.671	0.0389264	Down
	HMDB0014593	Droperidol	424.165	13.59	4.5164	0.663	0.0221951	Down
	CSID24680206	Setrobuvir	559.117	13.60	1.3963	0.659	0.0416337	Down
	CSID391610	L787257	687.079	13.60	1.3611	0.652	0.0374926	Down
	HMDB0041851	Cefpimizole	669.106	13.60	1.2637	0.617	0.0282558	Down
	CSID25977	12-O-Tetradecanoylphorbol-13-acetate	661.399	13.52	4.6158	0.602	0.0403694	Down
	HMDB0036329	Homocapsaicin	364.210	13.49	2.2724	0.571	0.0192239	Down
	CSID220457	acetate2?,3?-(difluoromethylene)-5?-androstan-17?-ol	347.220	13.49	7.6807	0.570	0.0373371	Down
	HMDB0004704	9,10-DHOME	627.450	13.51	3.1355	0.524	0.0394049	Down
	HMDB0010333	Estriol-17-glucuronide	445.190	5.49	1.8111	0.514	0.0252590	Down
	HMDB0061864	Dihomolinoleic acid	279.234	13.50	24.7971	0.486	0.0214515	Down
	HMDB0032783	Porrigenin A	895.634	13.51	3.6606	0.485	0.0384935	Down
	CSID10128403	4-Methyl-4-aza-5-pregnene-3,20-dione	328.225	13.49	1.4316	0.485	0.0248330	Down
	HMDB0007085	DG[15:0/20:5 (5Z,8Z,11Z,14Z,17Z)/0:0]	581.455	13.49	5.6945	0.476	0.0343696	Down
	HMDB0015397	Sunitinib	397.205	7.15	4.0719	0.444	0.0399618	Down
	CSID389265	N-benzoyl-D-arginine-4-nitroanilide	379.157	13.50	4.1639	0.391	0.0460232	Down
	CSID37751	triafungin	443.174	5.60	1.3673	0.350	0.0080564	Down
	HMDB0040608	Kanzonol F	465.192	7.15	1.1935	0.337	0.0272227	Down
	CSID142795	momilactone A	627.375	7.28	1.9674	0.306	0.0030993	Down
	CSID10706	Tineafax	429.194	5.93	1.4772	0.253	0.0126354	Down
	CSID82423	Roxatidine	611.380	8.27	1.0168	0.246	0.0050683	Down
	CSID388960	1-(beta-D-Ribofuranosyl)-1,2-dihydropyrimidine	427.179	5.96	1.2803	0.224	0.0022988	Down
	HMDB0011603	4-(Methylnitrosamino)-1-(3-pyridyl)-1-butanone	413.199	5.68	1.0836	0.210	0.0244314	Down
C vs. A	CSID2338563	CLONIXIN LYSINE	453.158	6.45	2.8602		0.0410226	Up
	HMDB0013636	Pyrroloquinoline quinone	329.000	6.67	5.4404		0.0188619	Up
	CSID142662	Furosemide sodium	396.987	6.67	2.1791	61302.968	0.0367756	Up
	CSID17021	ENTSUFON SODIUM	423.184	7.64	5.6277	949.957	0.0178690	Up
	HMDB0029510	Garcinone B	439.142	5.34	1.7964	453.719	0.0084223	Up
	CSID389476	(1R,4R,13R,14S)-13-Hydroxy-9-(1-hydroxyethyl)-3-[5-(3-hydroxyphenyl)-5-methoxy-2-pentanyl]-4,14,16,16-tetramethyl-2,6,10,17-tetraoxatricyclo[11.3.1.1∼1,5∼]octadecane-7,11-dione	591.318	8.39	1.1143	79.163	0.0124371	Up
	HMDB0062779	Cortisol 21-sulfate	441.158	4.79	1.3637	61.756	0.0201343	Up
	HMDB0041554	Artemisyl propionate	255.160	8.00	1.4197	25.256	0.0387194	Up
	HMDB0004158	D-Urobilinogen	589.302	5.72	2.0553	13.969	0.0285637	Up
	HMDB0034286	Ethyl 10-undecenoate	257.175	8.41	1.0486	3.665	0.0465114	Up
	CSID29271896	Liafensine	411.183	6.66	1.9145	3.126	0.0471294	Up
	CSID4939099	PYRANTEL (+)-TARTRATE SALT	355.098	1.26	1.0240	3.048	0.0300985	Up
	HMDB0039340	Ichangin 4-glucoside	631.241	7.80	1.9204	2.374	0.0176500	Up
	HMDB0010333	Estriol-17-glucuronide	445.189	5.28	1.1167	2.054	0.0073601	Up
	CSID390381	Bruceoside A	663.230	8.20	1.1202	1.905	0.0185666	Up
	HMDB0041480	(Z)-13-Octadecenoic acid	281.248	9.82	1.0403	1.467	0.0125231	Up
	HMDB0010333	Estriol-17-glucuronide	445.190	5.49	1.2568	1.456	0.0439706	Up
	HMDB0031078	Pentadecanal	271.228	10.03	1.0618	1.456	0.0098243	Up
	HMDB0030978	11-Oxohexadecanoic acid	269.212	11.11	1.2436	1.350	0.0019975	Up
	HMDB0112190	12-Hydroxyhexadecanoic acid	271.227	12.37	1.3358	1.209	0.0186780	Up
	HMDB0031039	Heptadecanal	299.259	11.34	1.8027	1.129	0.0321475	Up
	HMDB0011473	LysoPE(0:0/16:0)	452.278	10.01	3.3141	0.762	0.0398565	Down
	HMDB0012883	Adrenochrome o-semiquinone	180.067	1.16	2.3428	0.756	0.0327723	Down
	HMDB0010381	LysoPC(15:0)	480.308	11.09	1.0645	0.744	0.0239564	Down
	HMDB0011473	LysoPE(0:0/16:0)	452.277	9.79	1.1885	0.743	0.0450400	Down
	HMDB0010393	LysoPC[20:3 (5Z,8Z,11Z)]	590.346	9.92	1.4171	0.715	0.0152067	Down
	HMDB0011475	LysoPE[0:0/18:1 (11Z)]	478.293	9.24	1.2834	0.712	0.0375563	Down
	HMDB0060484	Indolepyruvate	248.054	1.16	1.2608	0.699	0.0036761	Down
	CSID2339128	Apramycin	520.265	10.01	1.2542	0.686	0.0172288	Down
	HMDB0010393	LysoPC[20:3 (5Z,8Z,11Z)]	590.347	10.10	4.6386	0.658	0.0028978	Down
	CSID29738718	Setipiprant	383.122	1.16	1.1363	0.494	0.0211778	Down

### 3.3 KEGG pathway analysis of DEMs

**Figure 4:**
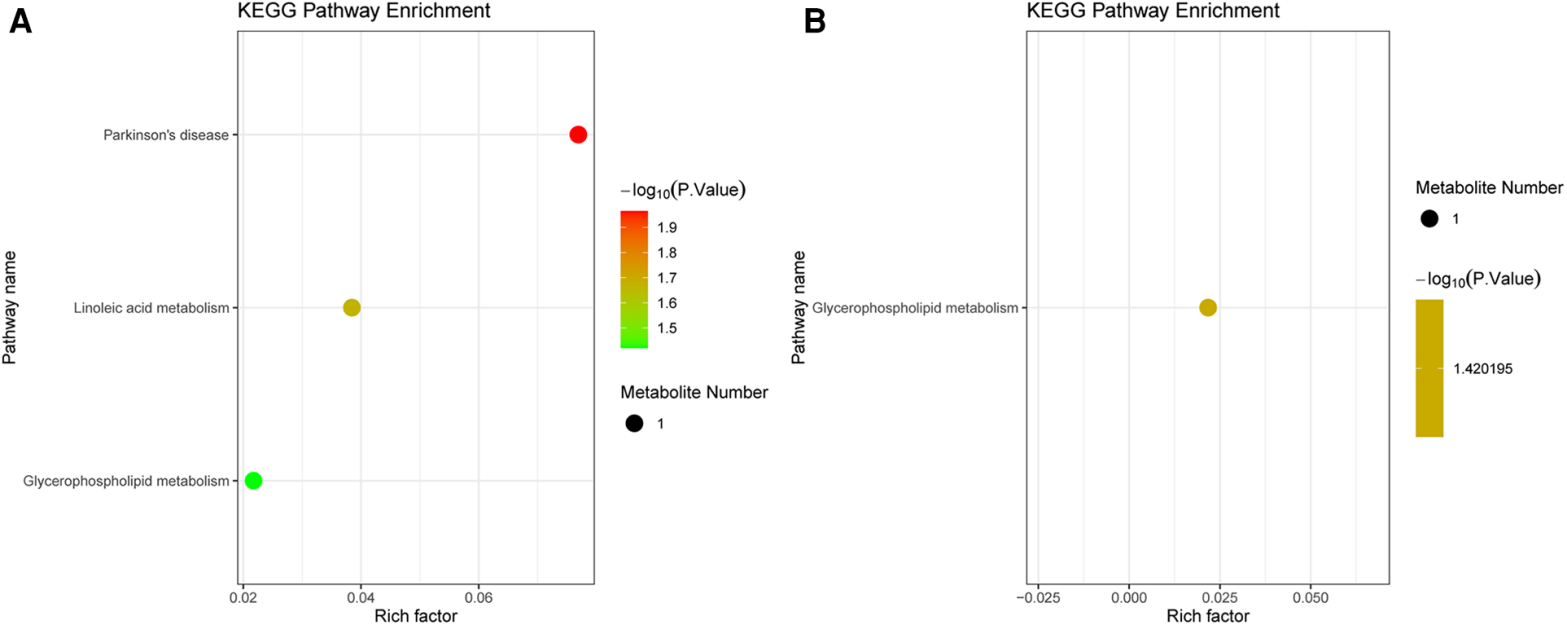
KEGG analysis in metabolomics. (**A**) Pathway enrichment of the DEMs between group B and A. (**B**) Pathway enrichment of the DEMs between group C and A. The dot color represents the *p*-value and the dot size represents the number of DEMs. DEMs, differentially expressed metabolites.

The DEMs were subjected to KEGG-enrichment analysis, and enriched pathways with significant differences were identified. The enriched metabolic pathways were Parkinson's disease, linoleic and metabolism and glycerophospholipid metabolism in group B compared to A (Figure 4A). The enriched metabolic pathway was glycerophospholipid metabolism in group C compared to A (Figure 4B). Compared with group A, the common enrichment metabolic pathway of group B and C was glycerophospholipid metabolism. Correlation analyses between transcriptomics and metabolomics.

**Figure 5:**
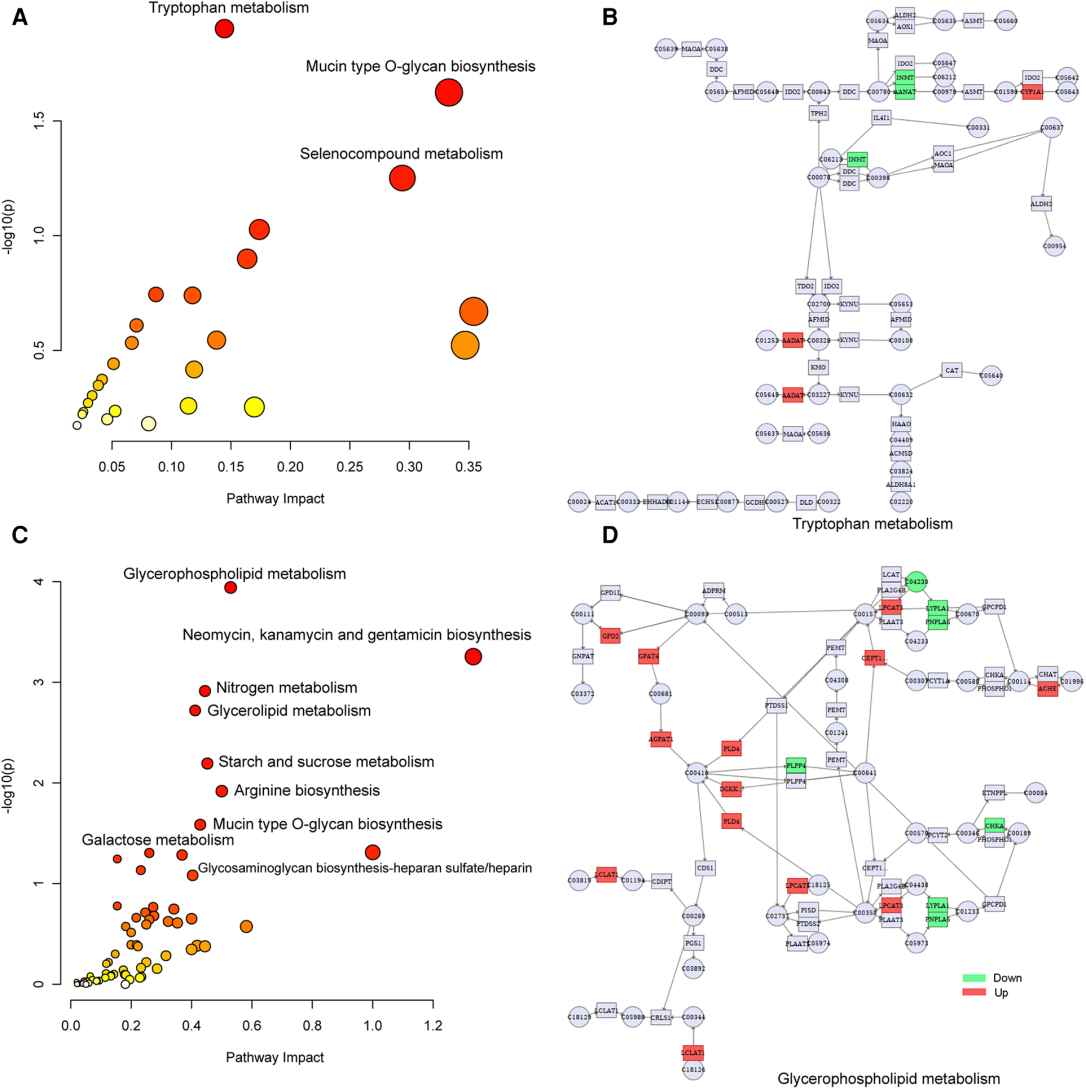
Joint-pathway analysis of DEGs and DEMs. (**A**) Joint-pathway analysis of DEGs and DEMs between group B and A. (**B**) Tryptophan metabolism pathway. (**C**) Joint-pathway analysis of DEGs and DEMs between group C and A. (**D**) Glycerophospholipid metabolism pathway. DEGs, differentially expressed genes; DEMs, differentially expressed metabolites.

**Figure 6:**
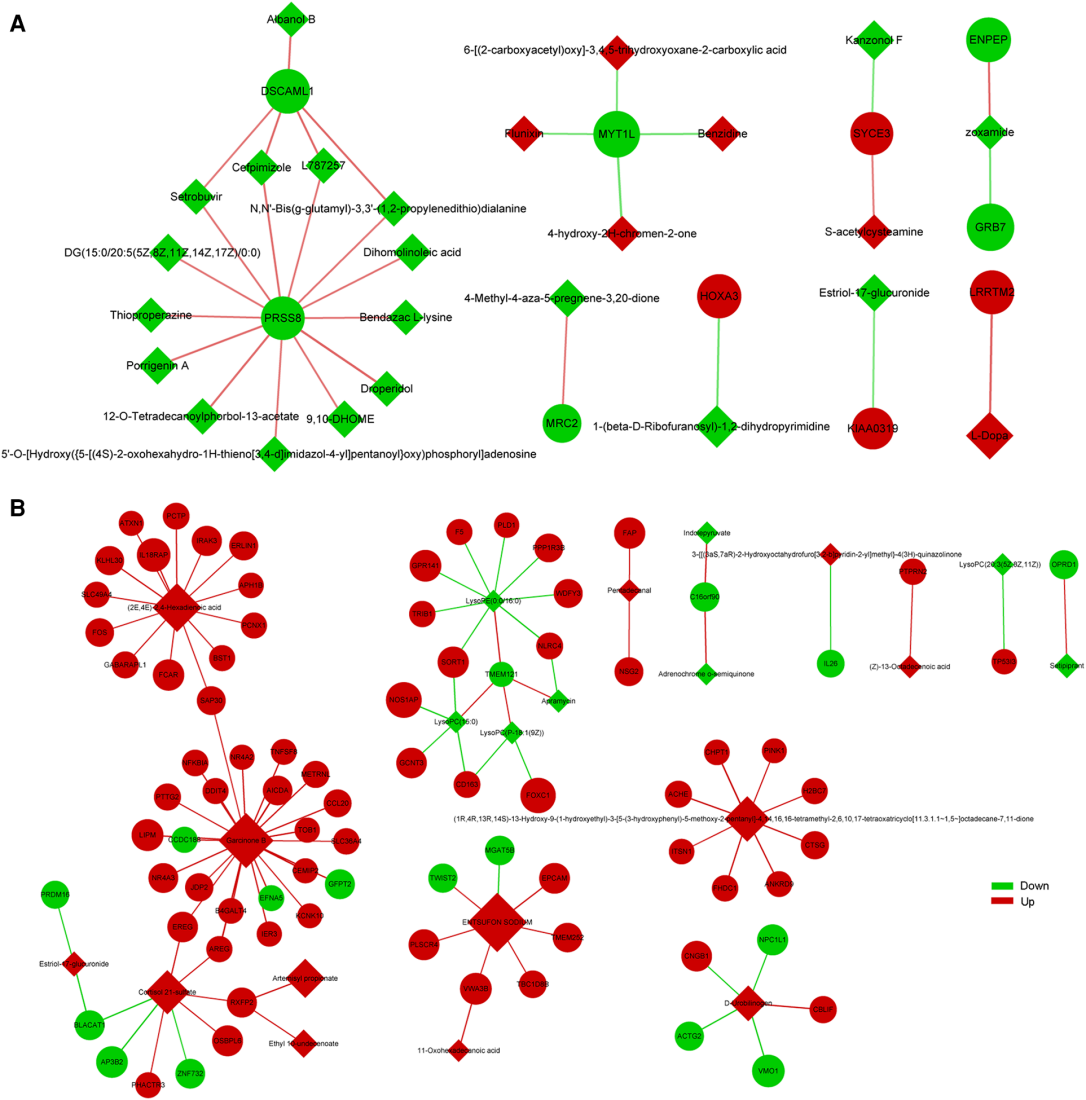
Correlation analysis of DEGs and DEMs between group B and A (**A**), group C and A (**B**) the quadrangle indicates metabolites, and the nodes indicate genes.

To explore the factors affecting nutritional risk and neurodevelopment in children with CHD by linking important metabolites and genes through shared metabolic pathways, joint pathway analysis between DEGs and DEMs were performed. The pathways are shown in Figures 5A,B, which includes tryptophan metabolism and mucin type O-glycan biosynthesis in group B compared to A. Nine significantly altered pathways were revealed in group C compared to A, include glycerophospholipid metabolism, neomycin, kanamycin and gentamicin biosynthesis, nitrogen metabolism, glycerolipid metabolism, starch and sucrose metabolism, arginine biosynthesis, mucin type O-glycan biosynthesis, glycosaminoglycan biosynthesis-heparan sulfate/heparin and galactose metabolism (Figures 5C,D). Correlation analysis utilized Pearson calculation to show the correlation of the DEMs and DEGs. Figure 6 showed strong correlations among transcripts and metabolites.

## 4 Discussion

In this study, we divided children with CHD into three groups based on nutritional risk and neurodevelopment, and integrated transcriptomics and metabolomics data to explore molecular mechanisms affecting nutritional risk and neurodevelopment in children with CHD. We found that NSUN7, SLC6A8, CXCL1 and LCN8 were significantly different in groups B compared to A, and SLC1A3 and LCN8 were significantly different in groups C compared to A. NSUN7, a m^5^C RNA methyltransferase gene, is differentially expressed in Alzheimer's disease patients (24). Solute carrier (SLC) are a group of membrane transport proteins that facilitate the transport of various substrates across biofilms (25). SLC6A8 could modulate human creatine levels and suppress colon cancer progression (26). Previous studies have shown that SLC6A8 can be up-regulated by p65/NF-κB transcription and mediate intracellular creatine accumulation in hypoxia (27). Creatine deficiency may be manifested as intellectual and behavioral disorders (28), so we suspect that SLC6A8 is related to neurodevelopment in children with CHD. SLC1A3 is an aspartate/glutamate transporter that maintains electron transport chain and tricarboxylic acid cycle activity (29). Studies have also shown that SLC1A3 may affect glucose metabolism by activating the PI3 K/AKT signaling pathway to provide energy for cell proliferation (30), which also seems to confirm its correlation with nutritional risk. Besides, SLC1A3 was also related to the development of stress and depression (31). CXCL1 was expressed in nervous systems and involved in the development of inflammation and pain (32). LCN8, a member of the liposome protein family, was found to be associated with the severity of post-traumatic stress disorder symptoms by genome-wide DNA methylation analysis (33). Therefore, we deduced that NSUN7, CXCL1, SLC6A8 and LCN8 may be involved in the neurodevelopment in children with CHD, SLC1A3 and LCN8 may be involved in the nutritional risk. We also found that cytokine receptor interaction was found to be the most altered pathway in both two groups compared to the control group. Cytokines play an important regulatory role in various processes, including immune function, inflammation, hematopoiesis, cell growth and differentiation (34). Cytokine receptor interaction provides a new perspective to explore the molecular mechanism affecting nutritional risk and neurodevelopment in children with CHD.

Metabolomics analysis found the levels of Albanol B, dihomo-linoleic acid, lysophosphatidylethanolamine (LysoPE) and lysophosphatidylcholine (LysoPC) were significantly altered in group B compared to A, and phospholipids and amino acids were significantly altered in group C compared to A. Albanol B, a compound isolated in root bark, could be involved in Anti-Alzheimer's disease (35). Dihomo-linoleic acid could drive ferroptosis-mediated neurodegeneration (36). LysoPE and LysoPC are lysophospholipids, a type of phospholipid. Lysophospholipids are involved in many physiological processes, including regulating neuropathic pain, disorders of neuroectodermal development, and disorders of neuroectodermal development (37, 38). Lipids are closely related to Alzheimer's disease (39, 40). Several key pathways were identified in group B compared to A (neurodevelopment disorder enriched comparison), including Parkinson's disease, linoleic acid metabolism and glycerophospholipid metabolism. Glycerophospholipid metabolism is also a critical pathway in group C compared to A. Linoleic acid is essential for optimal growth and brain development in infants and can regulate key neurodevelopmental processes (41). Studies have shown that cancer patients will suffer from malnutrition, and the increase of nutritional risk will increase the content of linoleic acid in fecal supernatant, indicating that linoleic acid is a biomarker of high nutritional risk (42). Linoleic acid can synthesize polyunsaturated fatty acids required by human brain cells, which is conducive to promoting brain development and plays a certain role in promoting the development of children's intelligence (41). This is consistent with our study that the linoleic acid metabolic pathway is associated with neurodevelopment in children with CHD. A basic study has demonstrated that dysregulation of glycerophospholipid and sphingolipid metabolism may have a negative impact on neurodevelopment in offspring, while lipid changes may disturb phospholipid homeostasis, altering membrane integrity, orientation, permeability, and function, leading to neurological dysfunction and degeneration (43). Choline is the main component of synthetic phospholipids, phosphatidylcholine and sphingomyelin, and plays an important role in neurogenesis and neural migration during fetal development (44). Gut microbiota plays an important role in the onset of depression, and metabolomics analysis showed that significant differences in glycerophospholipids and fatty acids metabolism between depressed mice and normal mice (45). Phospholipid metabolism plays an important role in energy metabolism throughout the body, and phospholipid synthesis is essential for normal development and health (46). It can be concluded that linoleic acid metabolism and glycerophospholipid metabolism may be involved in the nutritional risk and neurodevelopment of children with CHD.

In joint analysis, glycerophospholipid metabolism was found to be a key pathway in group C compared to A (nutritional risk enriched comparison), which was consistent with metabolomic results. In the network diagram of glycerophospholipid metabolism, the expression of GPD2 and AChE were up-regulation in group C compared to A, which was consistent with transcriptomic results. GPD2, a glycerol 3-phosphate dehydrogenase, was a component of the glycerol phosphate shuttle process that promotes glucose oxidation, the production of acetyl coenzyme A, the acetylation of histones, and the induction of genes encoding inflammatory mediators (47). AChE is a serine protease that hydrolyzes the neurotransmitter acetylcholine into acetate and choline, thus terminating neurotransmission (48). Tryptophan metabolism was found to be a key pathway in group B compared to A (neurodevelopment disorder enriched comparison). Tryptophan can be involved in the pathophysiology of different neuropsychiatric diseases through the serotonin pathway to produce serotonin as a neurotransmitter and melatonin as a neuromodulator (49). Tryptophan metabolism is directly or indirectly regulated by intestinal microorganisms, and its metabolites have immune, metabolic and neuroregulatory functions, which has become a therapeutic target for various diseases (50). Tryptophan metabolism pathways are also associated with symptoms and neurodevelopment in children with autism spectrum disorders (51), which was consistent with our results.

In summary, the content of metabolites and gene expression in blood and serum samples were analyzed to explore molecular mechanisms affecting nutritional risk and neurodevelopment in children with CHD. We further identified NSUN7, SLC6A8, CXCL1 and LCN8 as possible molecular markers associated with neurodevelopment and SLC1A3 and LCN8 were associated with nutritional risk. Linoleic acid metabolism, glycerophospholipid metabolism and tryptophan metabolism pathways were associated with neurodevelopment and glycerophospholipid metabolism pathway was associated with nutritional risk in children with CHD. By integration of metabolomics and transcriptomics data, our study provided the molecular basis for nutritional risk and neurodevelopment in children with CHD that could provide reference for clinical decision-making for individualized nutrition supply and demand in Chinese patients with CHD. This study also lays the groundwork for validation studies in longitudinal assessments of neurodevelopmental outcomes and preclinical investigations of therapeutic interventions targeting the identified molecular pathways. These findings need to be validated in large numbers of patients in the future and additional biological validation of the particular molecular processes in *in vitro* or *in vivo* investigations is necessary.

## Data Availability

The original contributions presented in the study are publicly available. This data can be found here: https://www.ncbi.nlm.nih.gov/geo/query/acc.cgi?acc=GSE267250, accession number: etmtqqaajzqrpeb.

## Supplementary material

Supplementary Figure S1 Heat map for correlation analysis between transcriptomic samples.

Supplementary Figure S2 QC sample correlation analysis and OPLS-DA analysis. QC sample correlation analysis in negative mode (**A**) and in positive mode (**B**). OPLS-DA analysis screening DEMs in negative mode (**C**) and in positive mode (**D**) between group B and A. OPLS-DA analysis screening DEMs in negative mode (**E**) and in positive mode (**F**) between group C and A. DEMs, differentially expressed metabolites.

## References

[1] Diab NS Barish S Dong W Zhao S Allington G Yu X Molecular genetics and Complex inheritance of congenital heart disease. Genes (Basel). (2021) 12(7):1020. 10.3390/genes12071020 34209044 PMC8307500

[2] Stiller B Grundmann S Höhn R Kari FA Berger F Baumgartner H. Adults with congenital heart disease—a new, expanding group of patients. Dtsch Arztebl Int. (2023) 120(12):195–202. 10.3238/arztebl.m2023.0006 36727545 PMC10264649

[3] Pierpont ME Brueckner M Chung WK Garg V Lacro RV McGuire AL Genetic basis for congenital heart disease: revisited: a scientific statement from the American Heart Association. Circulation. (2018) 138(21):e653–711. 10.1161/cir.0000000000000606 30571578 PMC6555769

[4] Su Z Zou Z Hay SI Liu Y Li S Chen H Global, regional, and national time trends in mortality for congenital heart disease, 1990–2019: an age-period-cohort analysis for the global burden of disease 2019 study. EClinicalMedicine. (2022) 43:101249. 10.1016/j.eclinm.2021.101249 35059612 PMC8760503

[5] Nattel SN Adrianzen L Kessler EC Andelfinger G Dehaes M Côté-Corriveau G Congenital heart disease and neurodevelopment: clinical manifestations, genetics, mechanisms, and implications. Can J Cardiol. (2017) 33(12):1543–55. 10.1016/j.cjca.2017.09.020 29173597

[6] Homsy J Zaidi S Shen Y Ware JS Samocha KE Karczewski KJ *de novo* mutations in congenital heart disease with neurodevelopmental and other congenital anomalies. Science. (2015) 350(6265):1262–6. 10.1126/science.aac9396 26785492 PMC4890146

[7] Stephen J Maddirevula S Nampoothiri S Burke JD Herzog M Shukla A Bi-allelic Tmem94 truncating variants are associated with neurodevelopmental delay, congenital heart defects, and distinct facial dysmorphism. Am J Hum Genet. (2018) 103(6):948–67. 10.1016/j.ajhg.2018.11.001 30526868 PMC6288279

[8] Verrall CE Patel S Travitz L Tchieu J Dale RC Kasparian NA Biological and structural phenotypes associated with neurodevelopmental outcomes in congenital heart disease. Transl Pediatr. (2023) 12(4):768–86. 10.21037/tp-22-687 37181016 PMC10167385

[9] Vagha K Taksande A Kenjale S Vagha J Varma A. Neurodevelopmental assessment in children with congenital heart disease by applying the denver developmental screening test 2: a prospective cross-sectional study. Cureus. (2023) 15(1):e33373. 10.7759/cureus.33373 36751205 PMC9897704

[10] Tsintoni A Dimitriou G Karatza AA. Nutrition of neonates with congenital heart disease: existing evidence, conflicts and concerns. J Matern Fetal Neonatal Med. (2020) 33(14):2487–92. 10.1080/14767058.2018.1548602 30608033

[11] Zhang M Wang L Huang R Sun C Bao N Xu Z. Risk factors of malnutrition in Chinese children with congenital heart defect. BMC Pediatr. (2020) 20(1):213. 10.1186/s12887-020-02124-7 32404077 PMC7218652

[12] Marino BS Lipkin PH Newburger JW Peacock G Gerdes M Gaynor JW Neurodevelopmental outcomes in children with congenital heart disease: evaluation and management: a scientific statement from the American Heart Association. Circulation. (2012) 126(9):1143–72. 10.1161/CIR.0b013e318265ee8a 22851541

[13] Morton SU Norris-Brilliant A Cunningham S King E Goldmuntz E Brueckner M Association of potentially damaging *de novo* gene variants with neurologic outcomes in congenital heart disease. JAMA Netw Open. (2023) 6(1):e2253191. 10.1001/jamanetworkopen.2022.53191 36701153 PMC9880793

[14] Martini S Beghetti I Annunziata M Aceti A Galletti S Ragni L Enteral nutrition in term infants with congenital heart disease: knowledge gaps and future directions to improve clinical practice. Nutrients. (2021) 13(3):932. 10.3390/nu13030932 33805775 PMC8002077

[15] Lim CYS Lim JKB Moorakonda RB Ong C Mok YH Allen JC The impact of pre-operative nutritional status on outcomes following congenital heart surgery. Front Pediatr. (2019) 7:429. 10.3389/fped.2019.00429 31709202 PMC6820300

[16] Yin X Bose D Kwon A Hanks SC Jackson AU Stringham HM Integrating transcriptomics, metabolomics, and GWAS helps reveal molecular mechanisms for metabolite levels and disease risk. Am J Hum Genet. (2022) 109(10):1727–41. 10.1016/j.ajhg.2022.08.007 36055244 PMC9606383

[17] Sun YV Hu YJ. Integrative analysis of multi-omics data for discovery and functional studies of complex human diseases. Adv Genet. (2016) 93:147–90. 10.1016/bs.adgen.2015.11.004 26915271 PMC5742494

[18] Schmidt DR Patel R Kirsch DG Lewis CA Vander Heiden MG Locasale JW. Metabolomics in cancer research and emerging applications in clinical oncology. CA Cancer J Clin. (2021) 71(4):333–58. 10.3322/caac.21670 33982817 PMC8298088

[19] Dong S Wu L Duan Y Cui H Chen K Chen X Metabolic profile of heart tissue in cyanotic congenital heart disease. Am J Transl Res. (2021) 13(5):4224–32. 34150010 PMC8205768

[20] Jin N Yu M Du X Wu Z Zhai C Pan H Identification of potential serum biomarkers for congenital heart disease children with pulmonary arterial hypertension by metabonomics. BMC Cardiovasc Disord. (2023) 23(1):167. 10.1186/s12872-023-03171-5 36991345 PMC10061882

[21] Su M Pan T Chen QZ Zhou WW Gong Y Xu G Data analysis guidelines for single-cell RNA-Seq in biomedical studies and clinical applications. Mil Med Res. (2022) 9(1):68. 10.1186/s40779-022-00434-8 36461064 PMC9716519

[22] Liu G Zeng X Wu B Zhao J Pan Y. RNA-Seq analysis of peripheral blood mononuclear cells reveals unique transcriptional signatures associated with radiotherapy response of nasopharyngeal carcinoma and prognosis of head and neck cancer. Cancer Biol Ther. (2020) 21(2):139–46. 10.1080/15384047.2019.1670521 31698994 PMC7012055

[23] Du B Zhang F Zhou Q Cheng W Yu Z Li L Joint analysis of the metabolomics and transcriptomics uncovers the dysregulated network and develops the diagnostic model of high-risk neuroblastoma. Sci Rep. (2023) 13(1):16991. 10.1038/s41598-023-43988-w 37813883 PMC10562375

[24] Knight HM Öz MD PerezGrovas-Saltijeral A. Dysregulation of RNA modification systems in clinical populations with neurocognitive disorders. Neural Regen Res. (2024) 19(6):1256–61. 10.4103/1673-5374.385858 37905873 PMC11467953

[25] Liu X. SLC family transporters. Adv Exp Med Biol. (2019) 1141:101–202. 10.1007/978-981-13-7647-4_3 31571165

[26] Kurth I Yamaguchi N Andreu-Agullo C Tian HS Sridhar S Takeda S Therapeutic targeting of SLC6A8 creatine transporter suppresses colon cancer progression and modulates human creatine levels. Sci Adv. (2021) 7(41):eabi7511. 10.1126/sciadv.abi7511 34613776 PMC8494442

[27] Li Q Liu M Sun Y Jin T Zhu P Wan X SLC6A8-mediated intracellular creatine accumulation enhances hypoxic breast cancer cell survival via ameliorating oxidative stress. J Exp Clin Cancer Res. (2021) 40(1):168. 10.1186/s13046-021-01933-7 33990217 PMC8120850

[28] Shen M Yang G Chen Z Yang K Dong H Yin C Identification of novel variations in SLC6A8 and GAMT genes causing cerebral creatine deficiency syndrome. Clin Chim Acta. (2022) 532:29–36. 10.1016/j.cca.2022.05.006 35588794

[29] Tajan M Hock AK Blagih J Robertson NA Labuschagne CF Kruiswijk F A role for P53 in the adaptation to glutamine starvation through the expression of SLC1A3. Cell Metab. (2018) 28(5):721–36.e6. 10.1016/j.cmet.2018.07.005 30122553 PMC6224545

[30] Xu L Chen J Jia L Chen X Awaleh Moumin F Cai J. Slc1a3 promotes gastric cancer progression via the Pi3k/akt signalling pathway. J Cell Mol Med. (2020) 24(24):14392–404. 10.1111/jcmm.16060 33145952 PMC7753768

[31] Ghosh M Ali A Joshi S Srivastava AS Tapadia MG. SLC1A3 C3590T but not BDNF G196A is a predisposition factor for stress as well as depression, in an adolescent eastern Indian population. BMC Med Genet. (2020) 21(1):53. 10.1186/s12881-020-0993-6 32171272 PMC7071583

[32] Jiang S Liang J Li W Wang L Song M Xu S The role of Cxcl1/Cxcr2 axis in neurological diseases. Int Immunopharmacol. (2023) 120:110330. 10.1016/j.intimp.2023.110330 37247498

[33] Mehta D Bruenig D Carrillo-Roa T Lawford B Harvey W Morris CP Genomewide DNA methylation analysis in combat veterans reveals a novel locus for PTSD. Acta Psychiatr Scand. (2017) 136(5):493–505. 10.1111/acps.12778 28795405

[34] Crisponi L Buers I Rutsch F. CRLF1 and CLCF1 in development, health and disease. Int J Mol Sci. (2022) 23(2):992. 10.3390/ijms23020992 35055176 PMC8780587

[35] Kuk EB Jo AR Oh SI Sohn HS Seong SH Roy A Anti-alzheimer’s disease activity of compounds from the root bark of Morus Alba L. Arch Pharmacal Res. (2017) 40(3):338–49. 10.1007/s12272-017-0891-4 28093699

[36] Sarparast M Pourmand E Hinman J Vonarx D Reason T Zhang F Dihydroxy-metabolites of dihomo-*Γ*-linolenic acid drive ferroptosis-mediated neurodegeneration. ACS Cent Sci. (2023) 9(5):870–82. 10.1021/acscentsci.3c00052 37252355 PMC10214511

[37] Kano K Aoki J Hla T. Lysophospholipid mediators in health and disease. Annu Rev Pathol. (2022) 17:459–83. 10.1146/annurev-pathol-050420-025929 34813354 PMC9641500

[38] Takagi Y Nishikado S Omi J Aoki J. The many roles of lysophospholipid mediators and Japanese contributions to this field. Biol Pharm Bull. (2022) 45(8):1008–21. 10.1248/bpb.b22-00304 35908884

[39] Ferré-González L Lloret A Cháfer-Pericás C. Systematic review of brain and blood lipidomics in Alzheimer’s disease mouse models. Prog Lipid Res. (2023) 90:101223. 10.1016/j.plipres.2023.101223 36871907

[40] Otoki Y Yu D Shen Q Sahlas DJ Ramirez J Gao F Quantitative lipidomic analysis of serum phospholipids reveals dissociable markers of Alzheimer’s disease and subcortical cerebrovascular disease. J Alzheimers Dis. (2023) 93(2):665–82. 10.3233/jad-220795 37092220

[41] Souza F dC Grodzki ACG Morgan RK Zhang Z Taha AY Lein PJ. Oxidized linoleic acid metabolites regulate neuronal morphogenesis *in vitro*. Neurochem Int. (2023) 164:105506. 10.1016/j.neuint.2023.105506 36758902 PMC10495953

[42] Ma CY Zhao J Qian KY Xu Z Xu XT Zhou JY. Analysis of nutritional risk, skeletal muscle depletion, and lipid metabolism phenotype in acute radiation enteritis. World J Gastrointest Surg. (2023) 15(12):2831–43. 10.4240/wjgs.v15.i12.2831 38222011 PMC10784828

[43] Jin Y Hu X Meng F Luo Q Liu H Yang Z. Sevoflurane exposure of clinical doses in pregnant rats induces vcan changes without significant neural apoptosis in the offspring. Medicina (Kaunas, Lithuania). (2023) 59(2):190. 10.3390/medicina59020190 36837392 PMC9965787

[44] Xia ZL Tan XY Song YY. Advances in basic research on choline and central nervous system development and related disorders. Zhonghua Yu Fang Yi Xue Za Zhi. (2023) 57(5):793–800. 10.3760/cma.j.cn112150-20220531-00548 37165829

[45] Tian T Mao Q Xie J Wang Y Shao WH Zhong Q Multi-omics data reveals the disturbance of glycerophospholipid metabolism caused by disordered gut microbiota in depressed mice. J Adv Res. (2022) 39:135–45. 10.1016/j.jare.2021.10.002 35777903 PMC9263645

[46] van der Veen JN Kennelly JP Wan S Vance JE Vance DE Jacobs RL. The critical role of phosphatidylcholine and phosphatidylethanolamine metabolism in health and disease. Biochim Biophys Acta Biomembr. (2017) 1859(9 Pt B):1558–72. 10.1016/j.bbamem.2017.04.006 28411170

[47] Langston PK Nambu A Jung J Shibata M Aksoylar HI Lei J Glycerol phosphate shuttle enzyme Gpd2 regulates macrophage inflammatory responses. Nat Immunol. (2019) 20(9):1186–95. 10.1038/s41590-019-0453-7 31384058 PMC6707851

[48] Shaikh S Verma A Siddiqui S Ahmad SS Rizvi SM Shakil S Current acetylcholinesterase-inhibitors: a neuroinformatics perspective. CNS Neurol Disord Drug Targets. (2014) 13(3):391–401. 10.2174/18715273113126660166 24059296

[49] Davidson M Rashidi N Nurgali K Apostolopoulos V. The role of tryptophan metabolites in neuropsychiatric disorders. Int J Mol Sci. (2022) 23(17):9968. 10.3390/ijms23179968 36077360 PMC9456464

[50] Gao K Mu CL Farzi A Zhu WY. Tryptophan metabolism: a link between the gut microbiota and brain. Adv Nutr. (2020) 11(3):709–23. 10.1093/advances/nmz127 31825083 PMC7231603

[51] Zhu J Hua X Yang T Guo M Li Q Xiao L Alterations in gut vitamin and amino acid metabolism are associated with symptoms and neurodevelopment in children with autism Spectrum disorder. J Autism Dev Disord. (2022) 52(7):3116–28. 10.1007/s10803-021-05066-w 34263410 PMC9213278

