# Supplementary material for: Transcriptomics integrated with metabolomics reveals partial molecular mechanisms of nutritional risk and neurodevelopment in children with congenital heart disease
Source: Front Cardiovasc Med. 2024 Aug 9;11:1414089. doi: 10.3389/fcvm.2024.1414089 (PMC11341388; doi:10.3389/fcvm.2024.1414089)
Supplement: Supplementary file 1 [file Table1.pdf]

Supplementary table 1 The annotation of GO analysis in figure 2Aand figure 2C

ID	Annotation
GO:0045087	innate immune response
GO:0009615	response to virus
GO:0051607	defense response to virus
GO:0045071	negative regulation of viral genome replication
GO:0061844	antimicrobial humoral immune response mediated by antimicrobial peptide
GO:0071222	cellular response to lipopolysaccharide
GO:0060700	regulation of ribonuclease activity
GO:0008584	male gonad development
GO:0070106	interleukin-27-mediated signaling pathway
GO:0070098	chemokine-mediated signaling pathway
GO:0005576	extracellular region
GO:0005615	extracellular space
GO:0005886	plasma membrane
GO:0005887	integral component of plasma membrane
GO:0016324	apical plasma membrane
GO:0048471	perinuclear region of cytoplasm
GO:0043025	neuronal cell body
GO:0045202	synapse
GO:0000786	nucleosome
GO:0070062	extracellular exosome
GO:0043236	laminin binding
GO:0001730	2’-5’-oligoadenylate synthetase activity
GO:0045236	CXCR chemokine receptor binding
GO:0004867	serine-type endopeptidase inhibitor activity
GO:0046982	protein heterodimerization activity
GO:0005515	protein binding

GO:0030527	structural constituent of chromatin
GO:0008009	chemokine activity
GO:0003779	actin binding
GO:0042803	protein homodimerization activity
GO:0006954	inflammatory response
GO:0007165	signal transduction
GO:0032731	positive regulation of interleukin-1 beta production
GO:0006955	immune response
GO:0019221	cytokine-mediated signaling pathway
GO:0016020	membrane
GO:0070821	tertiary granule membrane
GO:0016021	integral component of membrane
GO:0030667	secretory granule membrane
GO:0035579	specific granule membrane
GO:0009986	cell surface
GO:0035580	specific granule lumen
GO:0005829	cytosol
GO:0003953	NAD^+ nucleosidase activity
GO:0061809	NAD^+ nucleotidase, cyclic ADP-ribose generating
GO:0050135	$NAD(P)^+$ nucleosidase activity
GO:0019899	enzyme binding
GO:0038187	pattern recognition receptor activity
GO:0042802	identical protein binding
GO:0004888	transmembrane signaling receptor activity
GO:0005524	ATP binding
GO:0005102	receptor binding
