# Supplementary figures and images for: Transcriptomics integrated with metabolomics reveals partial molecular mechanisms of nutritional risk and neurodevelopment in children with congenital heart disease
Source: Front Cardiovasc Med. 2024 Aug 9;11:1414089. doi: 10.3389/fcvm.2024.1414089 (PMC11341388; doi:10.3389/fcvm.2024.1414089)

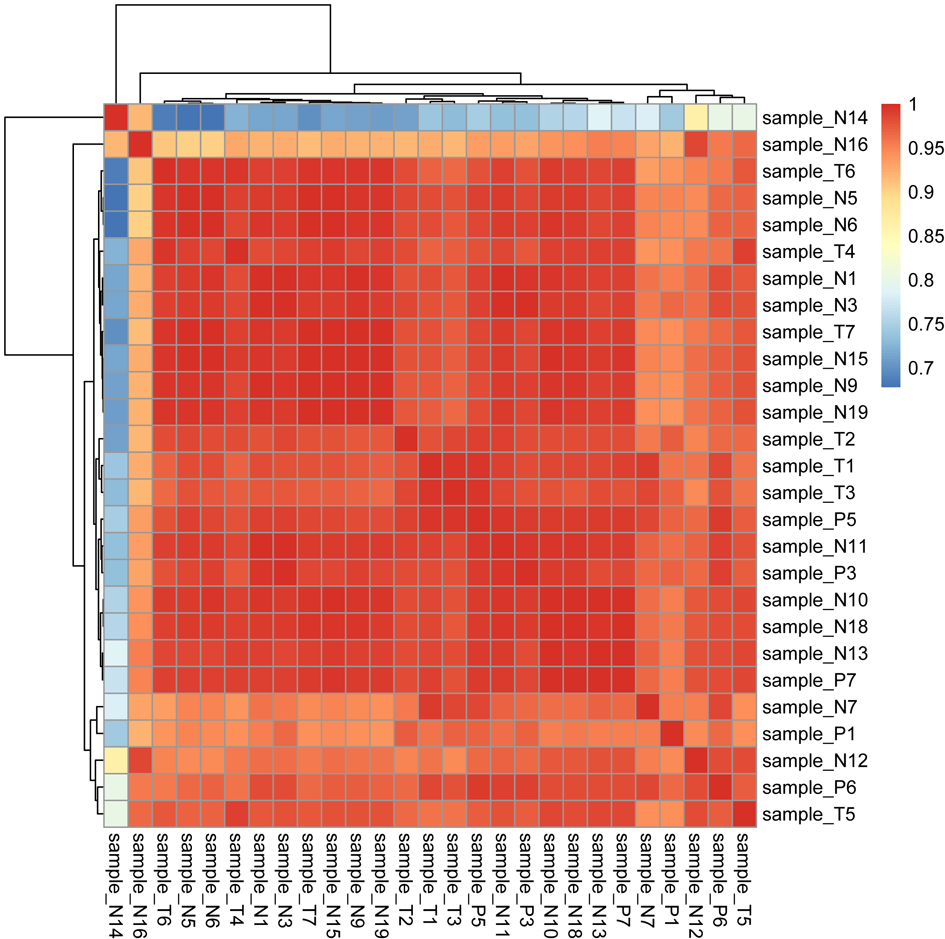

Supplement: Supplementary Figure S1 — Heat map for correlation analysis between transcriptomic samples. [file Image1.tif]

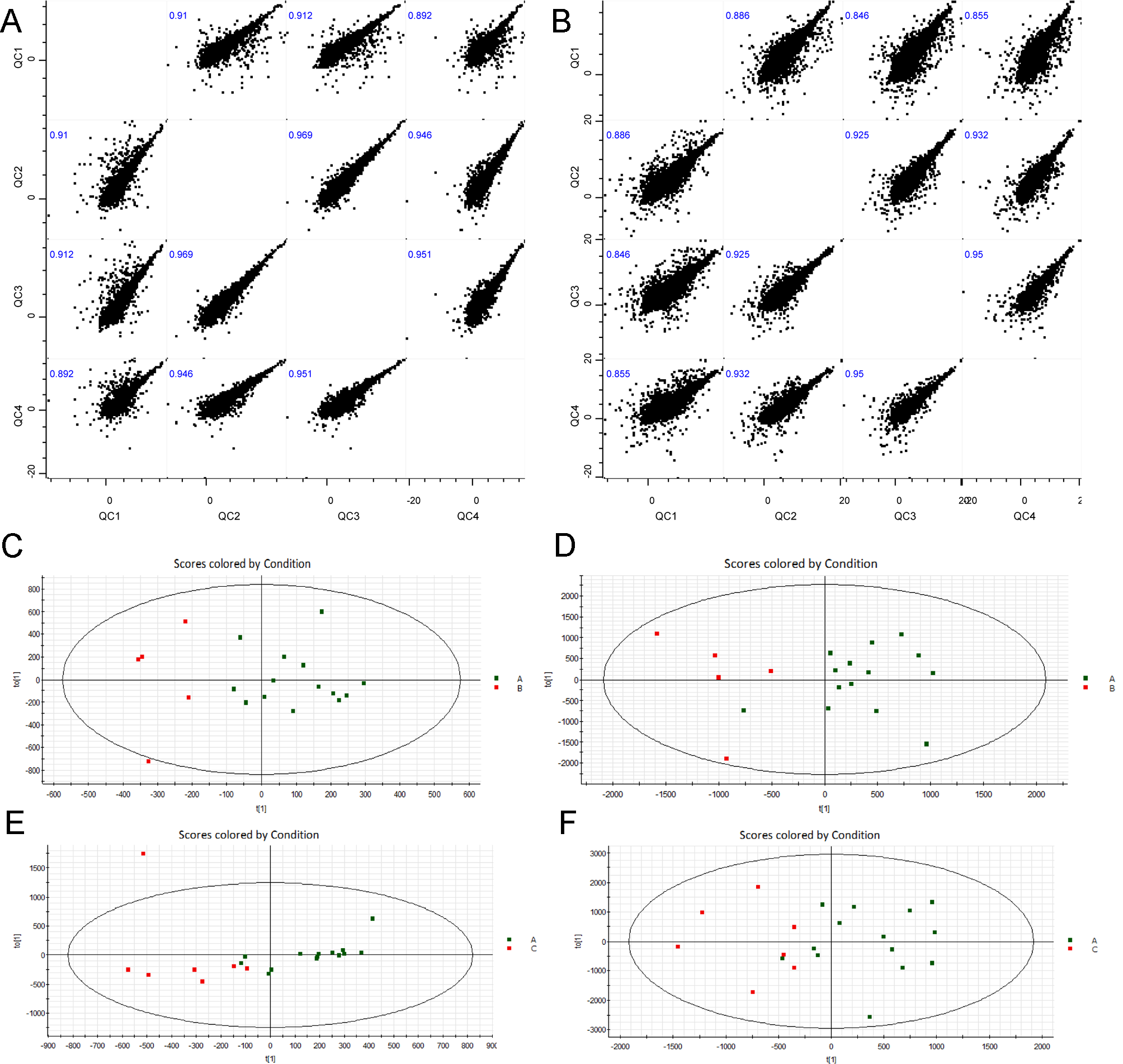

Supplement: Supplementary Figure S2 — QC sample correlation analysis and OPLS-DA analysis. QC sample correlation analysis in negative mode (A) and in positive mode (B). OPLS-DA analysis screening DEMs in negative mode (C) and in positive mode (D) between group B and A. OPLS-DA analysis screening DEMs in negative mode (E) and in positive mode (F) between group C and A. DEMs, differentially expressed metabolites. [file Image2.tif]
